# Gender differences in the association between cognitive social capital, self-rated health, and depressive symptoms: a comparative analysis of Sweden and Ukraine

**DOI:** 10.1186/s13033-016-0068-4

**Published:** 2016-05-04

**Authors:** Kateryna Karhina, Nawi Ng, Mehdi Ghazinour, Malin Eriksson

**Affiliations:** Department of Public Health and Clinical Medicine, Epidemiology and Global Health, Umeå University, 901 87 Umeå, Sweden; Police Education Unit, Umeå University, 901 87 Umeå, Sweden; Department of Social Work, Umeå University, 901 87 Umeå, Sweden

**Keywords:** Social capital, Trust, Safety, Self-rated health, Depressive symptoms, Sweden, Ukraine

## Abstract

**Background:**

Social capital is one of the social determinants of health, but there is still a lack of studies comparing its significance for health in different cultural settings. This study investigates and compares the relations between individual cognitive social capital and depressive symptoms and self-rated health in Sweden and Ukraine for men and women separately.

**Study design:**

Two cross-sectional nationally representative surveys of adult populations were used for the analysis. Data from the Ukraine’s World Health Survey and the Sweden’s National Public Health Survey were analyzed in this comparative study.

**Methods:**

The independent variable, cognitive social capital, was operationalized as institutional trust and feelings of safety. Depressive symptoms and self-rated health were used as the outcome variables. Crude and adjusted odds ratios and the 95 % confidence intervals were calculated using logistic regression. The model also adjusted for socio-demographic and lifestyle variables.

**Results:**

Institutional trust is higher in Sweden compared to Ukraine (31 % of the Swedes vs. 12 % of the Ukrainians reported high trust to their national government/parliament). There is a strong association between self-rated health and institutional trust for both sexes in Sweden (odds ratio/OR = 1.99; 95 % CI = 1.58–2.50 for women and OR = 1.82, CI = 1.48–2.24 for men who reported low institutional trust compared with those with high institutional trust) but only for women (OR = 1.88, CI = 1.12–3.15) in Ukraine. Trust thus seems to be more important for self-rated health of women and men in Sweden compared to their counterparts in Ukraine. Significant associations between depressive symptoms and institutional trust were not observed in either country after adjusting for socio-demographic and lifestyle factors. A lack of feeling of safety increased the odds of having depressive symptoms among women (OR = 1.97, CI = 1.41–2.76) and men (OR = 3.91, CI = 2.19–6.97) in Sweden. The same association was observed for poor self-rated health among Swedish women (OR = 2.15, CI = 1.55–2.99) and men (OR = 2.75, CI = 1.58–4.80). In Ukraine, a lack of feeling of safety did not show any significant association with self-rated health or depressive symptoms for men, but it increased the odds of depressive symptoms among women (OR = 1.72, CI = 1.13–2.62).

**Conclusions:**

In general, individual cognitive social capital is higher in Sweden than in Ukraine, and there is a stronger association between cognitive social capital and self-rated health in Sweden than in Ukraine. Interventions aiming to increase cognitive social capital for health promoting purposes might be favorable in Sweden, but this is not evidently the case in Ukraine.

**Electronic supplementary material:**

The online version of this article (doi:10.1186/s13033-016-0068-4) contains supplementary material, which is available to authorized users.

## Background

The global burden of mental illness is on the rise, and according to the World Health Organization (WHO) depression is predicted to be the second leading cause of global burden of disease by 2020 [1]. Mental illness is also associated with many other health conditions and lifestyle factors such as smoking, obesity, and hypertension, thus the overall global prevalence of mental illness is probably underestimated [2]. In addition, self-rated health (SRH)—a commonly used health indicator in epidemiological research—has been found to be a powerful predictor of mortality and morbidity (including depression) in various socio-cultural contexts [3–5]. SRH is believed to reflect indications of ill-health, such as psychosocial well-being, that are not normally included in medical examinations but that might still be indicative for mental illness [6]. A longitudinal study, designed to examine the associations between depressive outcomes (depression and depression treatment) and quality of life among patients of primary care facilities in six culturally different settings (Israel, Brazil, Australia, Barcelona, Russian Federation, USA) found a consistent pattern in which poor SRH was associated with depressive symptoms across countries [7]. A study from Australia also found that poor SRH predicted the risk for future long-term depression among a primary-care cohort with depressive symptoms [4].

Despite an overall high global burden of depression, rates of it vary markedly between different countries, suggesting the importance of macro-scale social factors [1]. In addition, depression is almost always reported to be twice as common in women compared with men across diverse societies and social contexts, including Ukraine and Sweden [8]. A similar gendered pattern has also been found for SRH where men are more likely to report good SRH compared to women worldwide [9, 10]. Evidently, gender is an important determinant of both physical and mental health [1].

The WHO Commission on Social Determinants of Health highlighted the need for targeting the social determinants of health in order to improve health and to decrease social inequalities in health [11]. Social capital is perceived as one important social determinant of health that has received considerable attention within the fields of public health and epidemiology during the last decades. There are several definitions of social capital but they all include three main elements: social networks, norms of reciprocity and trust. Numerous studies support a positive association between social capital and physical health, and not least SRH [12–14]. This has been found in studies from different cultural contexts such as Belgium [15], Canada [16], Finland [17–19], Japan [20–22], Russia [23], Sweden [24, 25], Taiwan [26], the UK [27, 28], and the US [29]. In addition, a recent study from Japan reports that neighborhood social capital decreases physical abuse of infants among the mothers [30]. However, studies have also indicated that the association between social capital and SRH differs across countries. Poortinga [14] used data from the European Social Survey (including 22 countries) and found a positive association between individual social capital and good SRH in countries with high levels of social capital, while the same was not always true in countries with lower levels of social capital [14]. In addition, there might be gender differences in the associations between social capital and SRH. In their investigation among populations in 50 countries, Elgar et al. concluded that women might benefit more from social capital than men [31]. Similar gender differences in the association between social capital and SRH have been found in studies from the UK [32] and Sweden [33]. Studies on social capital and mental health have mainly been conducted in Western societies, including Sweden [34–38], while evidence from developing and transitional countries such as Ukraine is largely missing.

Social capital has been conceptualized as both an individual and a collective feature. Within the collectivist approach, social capital is described as a collective feature that characterizes geographical areas in terms of levels of trust, reciprocity, and civic engagement. One of the most utilized definitions within this approach to social capital is Robert Putnam’s, which defines social capital as “*trust, norms, and networks that can improve the efficiency of society by facilitating coordinated actions*” [39]. In this paper, we conceptualize social capital as an individual resource, i.e. as a resource that is available for individuals through their social networks involvement. Definitions within this “social network approach” to social capital emanate from sociology, and one commonly used definition is that of Alejandro Porte, who defines social capital as “*the ability of actors to secure benefits by virtue of membership in social networks or other social structures*” [40]. Individual social capital is believed to influence individual’s health in different ways by influencing psychosocial processes and reducing stress, by affecting access to health services and facilities, and by influencing health-related behaviors and choices [41, 42].

Social capital is a broad concept, and it comes in different forms. There is a distinction between structural and cognitive forms of social capital, which might have special importance for health outcomes. A recent systematic review by Ehsan and De Silva concluded that individual cognitive social capital has a protective effect against common mental disorders, but the same effect was not found for structural social capital [37]. Structural social capital includes people’s actions, in other words, their active participation in formal and informal social networks. Cognitive social capital describes the values and perceptions of people (i.e. what people feel with regards to their social network involvement) [43]. Cognitive social capital includes norms, reciprocity, values, altruism, and responsibility [42]. In addition, a feeling of safety is commonly used as an indicator for cognitive social capital.

In both Sweden and Ukraine, mental health problems are considered as a main public health issue [44, 45], but comparable figures are not easy to find [8]. Further, despite the fact that both Sweden and Ukraine are located in Europe, they have different histories and political structures, which might influence health as well as the growth and use of social capital in these countries. Using the typology of Esping-Andersen, Rostila [46] considers Sweden to be a social-democratic regime characterized by relatively good social benefits and high levels of social security. Following the same typology, Ukraine is considered a post-socialist regime and is characterized by lower levels of benefits and social security. Another significant difference between Sweden and Ukraine concerns gender equality. According to the Gender Inequality Index from 2013, Sweden is ranked as number four in the world when it comes to gender inequality, while Ukraine is ranked 61 out of 187 countries [47].

In this current study, we define gender as a set of relations used as an organizing principle of society. Thus, we follow the definition of Connell in stating that gender is a structure of social relations that builds on the perceptions of differences between males and females that are reflected in everyday social practices [48].

In order to rule out the policy implications for using social capital as a tool for public health and health promotion, more comparative research is needed [49]. More knowledge is needed about how social capital operates and is associated with health—especially mental health—among men and women in various socio-political contexts. It might be that some forms of social capital are more important for health in some context than others [50]. For instance, it has been shown that high levels of trust are more important for general well-being in high and middle income countries compared to low income countries. Equally, Habibov and Afandi compared the associations between self-rated health and social capital between the three post-socialist countries Armenia, Azerbaijan and Georgia and found a beneficial effect of social capital at area and individual levels in Georgia while only on individual level in the other countries [51]. However, to our knowledge there are few studies that have compared the association between social capital and health between Sweden, a country representing a social-democratic welfare regime and countries representing post-socialism regimes, such a Ukraine.

The overall aim of this study is to analyze and compare the associations between individual cognitive social capital (institutional trust and feeling of safety), SRH, and depressive symptoms among men and women in Sweden and Ukraine.

## Data and methods

### Study design and data

This study used secondary data from Ukraine’s World Health Survey (WHS) and the Swedish National Public Health Survey (SNPHS). The WHS is a nationally representative survey of the adult population 18 years and older that was initiated by the WHO in order to gather information about the populations’ health and health systems. Specially trained interviewers collected the data. The WHS survey have been pre-tested by WHO in a Multi-Country Survey Study (implemented in 2000–2001 in nationally representative populations in order to improve the methodologies in a systematic, standardized and comparable manner). Each step of the WHS involved certification of quality according to WHS Quality Assurance Standards and Guidelines [52]. The Ukraine’s 2003 WHS data was used, which consists of 2800 randomly sampled individuals 18 and older who participated in the survey. Sweden also took part in WHS, but comparisons with Ukraine were not possible to make based on those data since Sweden used the short format of the survey. The SNPHS is a nationally representative Swedish health survey. It was introduced in 2004 and has since been collected annually in Sweden. The questions for SNPHS were tested in a pilot study and then the construct validity of each question was tested by Statistics Sweden and the results modified the questions later [53]. The data from the first survey in 2004 was used in order to have comparable data with Ukraine’s 2003 WHS’ data. A total of 12,166 randomly selected adult individuals (aged 18–84 years) participated in this postal survey in 2004.

#### Outcome variables

The SRH and depressive symptoms were used as the outcome variables. The variables that were used to construct these outcome variables in both datasets, as well as how responses to each question were categorized in this study, are presented in Table 1.

**Table 1:** Questions and responses used to measure depressive symptoms and self-rated health in Sweden and Ukraine

Sweden			Ukraine		
	Good	Poor		Good	Poor
Self-rated health					
How do you assess your general state of health?	Very good Good	Fair Poor Very poor	In general, how would you rate your health today?	Very good Good	Moderate Bad Very bad
	No/low	Yes		No/low	Yes
Depressive symptoms					
1. Have you been able to concentrate on everything you have done for the last few weeks?	Better than usual As usual	Worse than usual Much worse than usual	1. Overall in the last 30 days, how much difficulty did you have with concentrating or remembering things?	None Mild	Moderate Severe Extreme
2. Did you feel unhappy and depressed in recent weeks?	Not at all No more than usual	More than usual Much more than usual	2. Overall in the last 30 days, how much of a problem did you have with feeling sad, low or depressed?	None Mild	Moderate Severe Extreme
3. Do you have one/some of the following disorders or symptoms? Insomnia	No	Yes, light form Yes, a lot of trouble	3. Overall in the last 30 days, how much of a problem did you have with sleeping, such as falling asleep, waking up frequently during the night, or waking up too early in the morning?	None Mild	Moderate Severe Extreme
4. Were you able to deal with all of your problems over the last few weeks?	Better than usual As usual	Worse than usual Much worse than usual	4. How often have you found that you could not cope with all the things that you had to do?	Never Almost never	Sometimes Fairly often Very often

#### Depressive symptoms

In the Ukrainian dataset, the response categories “none” and “mild” were combined to indicate no/low depressive symptoms for questions one (difficulties with concentrating and remembering), two (feeling low, sad, or depressed), and three (problems with sleeping). For the fourth question (inability to cope), the categories “never” and “almost never” were combined to indicate no/low depressive symptoms. The remaining categories in each of the questions were combined to indicate the presence of depressive symptoms.

In the Swedish dataset, the response categories “better than usual” and “as usual” for questions one (able to concentrate) and four (able to deal with all problems) were combined to indicate no/low depressive symptoms. For question two (feeling unhappy or depressed), the categories “not at all” and “no more than usual” were combined to indicate no/low depressive symptoms, and for question three the response “no” was used to indicate the absence of depressive symptoms. The remaining two categories in each of the questions were combined to indicate the presence of depressive symptoms.

No/low depressive symptoms for all four questions was categorized as having no/low depressive symptoms, while depressive symptoms on at least one of the variables was categorized as having depressive symptoms.

#### Self-rated health

The second outcome variable “*Self*-*rated health*” was constructed from responses to the questions “In general, how would you rate your health today?” (Ukraine) and “How do you assess your general state of health?” (Sweden). Both questions had a five-point response scale, and in this study “good” and “very good” health were combined to indicate “good” SRH, while the three remaining categories were combined to indicate “poor” SRH.

#### Cognitive social capital measure

The focus in this study was on individual cognitive social capital, which was operationalized as institutional trust and feeling of safety. Questions regarding institutional trust for both countries can be found in Table 2. Trust in the highest institutional political entity was the main interest and because only two questions represented the highest level of political structure, these were chosen to measure institutional trust in both countries.

**Table 2:** Questions used to measure individual cognitive social capital in Sweden and Ukraine

Sweden	Categories	Ukraine	Categories
Institutional trust			
How confident are you of the following institutions in the society? Parliament		How much of the time do you think you can trust the national government to do what is right?	
Very much	High	Almost always	High
Relatively much		Most of the time	
Not very much	Moderate	Some of the time	Moderate
Not at all	Low	Hardly ever	Low
Have no opinion	No opinion	Never	
Feeling of safety			
How safe and secure do you feel when you walk alone in your neighborhood when it is dark?		How safe do you feel when walking down your street alone after dark?	
Very safe	High	Completely safe	High
Pretty safe		Very safe	
Somewhat unsafe	Moderate	Moderately safe	Moderate
Very unsafe	Low	Slightly safe	Low
Never alone when it is dark	Never alone	Not safe at all	

The final categories of institutional trust were combined and categorized as “high”, “moderate”, and “low” for Sweden and Ukraine. In the Ukrainian dataset, those who answered “almost always” and “most of the time” were categorized as having “high” trust, while those who responded “some of the time” were categorized as having “moderate” trust, and those who responded “hardly ever” and “never” were categorized as having “low” trust. The Swedish dataset was recoded in a similar way, with the responses “very much” and “relatively much” categorized as having high trust, “not very much” as moderate trust, and “not at all” as having low trust. The “have no opinion” response was treated separately in the Swedish data because it was not comparable with any of the responses in the Ukrainian dataset.

The feeling of safety question was similar in both datasets: “How safe and secure do you feel when walking alone at your neighborhood when it is dark?” (Table 2). The response categories were recoded and combined and categorized as high, moderate, and low feeling of safety in both datasets. The “completely safe” and “very safe” categories were merged to create the “high” feeling of safety category in the Ukrainian dataset, “moderately safe” was recoded to “moderate”, and “slightly safe” and “not safe at all” were recoded to “low” feeling of safety. The Swedish dataset was categorized in the same way. In addition, the “never alone outside when it is dark” response in the Swedish data was treated separately because there was no similar category in the Ukrainian dataset.

#### Other independent variables

A number of variables such as age, education, having small children at home, marital status, daily smoking, and alcohol use were included as potential confounders in the analysis. These variables were chosen to represent socio-demographic situation, family structure, and lifestyle habits that could possibly have an effect on depressive symptoms, SRH, and social capital.

Age was categorized into 18–29, 30–59, and 60 years and older in both countries. Education was divided into “short”, “medium”, and “long”. “Short” includes education up until completed secondary school (or up to eight school years not including preschool); “medium” education includes completed high school (up to 10 school years not including preschool) or its equivalent and “long” education includes all education beyond completed high-school or equivalent. The variable “Having small children at home” somewhat differed between the two countries. In the Swedish data the question captured having children of 0–6 years old at home, while in the Ukrainian data the question captured having children up to 5 years old at home. Marital status was measured in both countries as living together with a partner (cohabiting) but not necessarily being officially married, and the categories were “living without a partner” and “living with a partner”. Smoking was measured as daily smoking (“yes” and “no”) in both datasets. Alcohol consumption was measured by the question “Have you ever tried alcohol?” in the Ukrainian data and “How often did you drink alcohol during the last 12 months?” in the Swedish dataset. These two questions were not directly comparable, but because it was not possible to find exactly similar and comparable questions in the datasets, and taking into account the importance of the relationship of alcohol intake and mental health problems, it was decided to include this variable in the analysis. Negative answers to both questions were categorized as “no”, while all other answers were coded as “yes” in order to indicate alcohol consumption ever.

## Statistical analysis

Descriptive statistics were calculated to estimate and compare the distribution of socio-demographic and lifestyle variables, access to cognitive social capital, and the distribution of depressive symptoms and SRH for men and women in Sweden and Ukraine. Binary logistic regression was conducted to analyze the associations between access to cognitive social capital and SRH and depressive symptoms independently. Sex-stratified binary logistic regression was conducted in both of the datasets. The results were presented as the odds ratio (OR) with 95 % confidence interval (CI). We have presented the results of the crude models (Additional files 1, 2: Tables S1, S2) and for models adjusted for other potential confounders. Stata 13 was used for the analyses.

## Results

### Descriptive statistics

Table 3 presents the distribution of socio-demographic and lifestyle variables, access to cognitive social capital, and distribution of depressive symptoms and SRH in Sweden and Ukraine, both overall and stratified by sex.

**Table 3:** Distribution of socio-demographic, lifestyle, social capital, and outcome variables of self-rated health and depressive symptoms in Sweden and Ukraine

Variables	Sweden			Ukraine		
	Women (%)	Men (%)	Total (%)	Women (%)	Men (%)	Total (%)
	N = 5436	N = 4612	N = 10,048	N = 1723	N = 910	N = 2633
Socio-demographic						
Age						
18–29	18.9	17.0	18.0	18.8	23.3	20.3
30–59	56.0	54.5	55.4	50.7	51.9	51.1
60+	25.1	28.6	26.6	30.5	24.8	28.6
Education						
Short	26.5	25.1	25.8	14.1	10.7	13.0
Medium	33.1	40.6	36.5	49.0	51.4	49.9
Long	40.4	34.3	37.7	36.8	37.9	37.2
Marital status						
Living without a partner	34.2	31.2	32.7	51.1	32.0	44.5
Living with partner	65.8	68.8	67.3	48.9	68.0	55.5
Small children at home						
Yes	13.7	12.8	13.3	11.6	8.5	10.5
No	86.3	87.2	86.7	88.4	91.5	89.5
Lifestyle						
Smoking						
Yes	18.0	13.4	15.8	6.7	43.8	19.6
No	82.0	86.6	84.2	93.3	56.2	80.4
Alcohol consumption ever						
Yes	86.3	92.0	88.9	69.3	84.3	74.4
Never	13.7	8.0	11.1	30.7	15.7	25.6
Social capital						
Trust in the national government/parliament						
High	30.7	31.6	31.1	11.7	11.6	11.7
Moderate	34.3	38.0	36.0	31.1	32.6	31.6
Low	9.4	17.0	12.9	57.4	55.7	56.8
No opinion	25.6	13.4	20.0			
Feeling of safety						
High	25.8	56.3	39.8	14.9	31.8	20.7
Moderate	62.1	40.7	52.2	50.0	52.0	50.6
Low	3.5	1.2	2.4	35.2	16.3	28.6
Never alone	8.6	1.8	5.5			
Mental and health outcomes						
Depressive symptoms						
No	78.5	85.8	81.8	62.2	79.8	68.3
Yes	21.5	14.2	18.2	37.8	20.2	31.7
Self-rated health						
Good	67.3	71.2	69.1	23.0	36.9	27.8
Poor	32.7	28.8	30.9	77.0	63.1	72.2

A greater proportion of respondents had long education in Sweden (38 %), while there were more people with medium education in Ukraine (50 %). A higher proportion of respondents were living without a partner in Ukraine than in Sweden (45 versus 33 %), and in both countries more women than men were living without a partner (34 versus 31 % in Sweden and 51 versus 32 % in Ukraine).

Sweden had a higher proportion of women who smoke compared to men (18 versus 13 %) while in Ukraine smoking was more prevalent among men (7 versus 44 %). The proportion of respondents who ever consumed alcohol was higher in Sweden compared to in Ukraine (89 versus 74 % respectively).

There were considerable differences in the level of institutional trust among the Swedish and Ukrainian respondents. A majority (57 %) of the Ukrainians had low level of institutional trust, while only 13 % of the Swedes reported a low level of institutional trust. While the levels of institutional trust were quite similar among Ukrainian men and women, there was a gendered pattern in Sweden such that a higher proportion of men had low institutional trust compared to women (17 compared to 9 %).

There were large differences in the feeling of safety among men and women in both countries. In both countries a much lower proportion of women reported feeling safe when walking outside in their neighborhoods after dark compared to men (26 versus 56 % in Sweden and 15 versus 32 % in Ukraine). However, there were also large differences between the countries. In Sweden, 56 % of men reported a high feeling of safety compared to only 32 % of men in Ukraine. The corresponding figures were 26 % for Swedish women and 15 % for Ukrainian women.

Depressive symptoms were more prevalent for women than men in both countries, but the proportion of both women and men with depressive symptoms was higher in Ukraine: 18 % in Sweden versus 32 % in Ukraine. The proportion of women and men in Sweden reported depressive symptoms was 22 versus 14 %, while the corresponding figures for women and men in Ukraine were 38 and 20 %, respectively.

The proportion of people who considered their health to be poor was considerably higher in Ukraine (72 %) compared to Sweden (31 %). In both countries, a higher proportion of women reported their health as poor compared to men (33 versus 29 % in Sweden and 77 versus 63 % in Ukraine).

### Association between depressive symptoms, social capital, and socio-demographic and lifestyle factors

Additional file 1: Table S1 presents the crude ORs for depressive symptoms for women and men in both countries by levels of social capital, socio-demographic, and lifestyle variables. There was no significant association between depressive symptoms and institutional trust in Ukraine. On the contrary, among Swedish men, having low trust significantly increased the odds of having depressive symptoms (OR = 1.44; 95 % CI = 1.14–1.83).

Feeling of safety was significantly associated with depressive symptoms among both Swedish women and men. The association was stronger for Swedish men than women, and for both sexes there was a gradient in that low level of the feeling of safety increased the odds for depressive symptoms more than having moderate-level of the feeling of safety. In Ukraine, no association between depressive symptoms and the feeling of safety was found for men, while for women the odds for depressive symptoms were higher for those who experience low level of feeling safety (1.62; 1.12–2.35) compared to women feeling a high level of safety. Though not statistically significant, this association was stronger for Swedish compared to Ukrainian women (2.19; 1.59–3.01).

Table 4 presents the adjusted OR for depressive symptoms by levels of cognitive social capital for women and men in both countries. After adjusting for socio-demographic and lifestyle factors, there were no significant associations between institutional trust and depressive symptoms among Swedish and Ukrainian women and men.

**Table 4:** Adjusted odds ratio (with 95 % confidence intervals) for depressive symptoms by levels of cognitive social capital for women and men in Sweden and Ukraine

Variables	Sweden		Ukraine	
	Women	Men	Women	Men
Social capital				
Trust in the national government/parliament				
High	1*	1*	1***	1***
Moderate	1.11 (0.94–1.32)	1.06 (0.86–1.30)	0.98 (0.62–1.54)	1.56 (0.70–3.49)
Low	1.25 (0.98–1.60)	1.27 (0.99–1.63)	1.20 (0.78–1.87)	1.69 (0.79–3.63)
No opinion	1.07 (0.89–1.30)	0.97 (0.73–1.30)		
Feeling of safety				
High	1***	1***	1***	1***
Moderate	1.52 (1.24–1.87)	3.00 (2.10–4.28)	1.04 (0.65–1.65)	0.93 (0.52–1.67)
Low	1.97 (1.41–2.76)	3.91 (2.19–6.97)	1.72 (1.13–2.62)	1.17 (0.69–1.98)
Never alone	1.95 (1.50–2.54)	3.21 (1.83–5.63)		
Other variables				
Age				
18–29	1***	1***	1***	1***
30–59	0.63 (0.53–0.75)	0.92 (0.73–1.16)	2.81 (1.85–4.26)	2.01 (0.94–4.30)
60+	0.27 (0.21–0.34)	0.49 (0.36–0.66)	8.66 (5.36–14.0)	6.15 (2.89–13.1)
Education				
Short	1***	1***	1***	1***
Medium	1.07 (0.86–1.34)	1.22 (0.94–1.58)	0.65 (0.40–1.06)	0.52 (0.27–0.99)
Long	1.22 (1.00–1.50)	1.24 (0.96–1.61)	0.47 (0.28–0.79)	0.65 (0.33–1.29)
Marital status				
Living without a partner	1***	1***	1***	1***
Living with partner	0.71 (0.62–0.73)	0.62 (0.51–0.75)	0.81 (0.60–1.08)	0.98 (0.58–1.66)
Small children				
No	1***	1***	1**	1**
Yes	1.26 (1.03–1.54)	1.53 (1.20–1.96)	1.50 (0.95–2.37)	1.41 (0.57–3.49)
Smoking				
No	1***	1***	1***	1***
Yes	1.12 (0.94–1.33)	1.51 (1.21–1.90)	1.03 (0.59–1.79)	0.89 (0.56–1.43)
Alcohol ever				
No	1	1	1**	1**
Yes	0.95 (0.77–1.1)	0.89 (0.65–1.20)	1.46 (1.05–2.02)	0.81 (0.45–1.46)

The asterisks denote significant level of Chi Square test of p < 0.05 (*) or p < 0.01 (**) or p < 0.001 (***)

The association between depressive symptoms and low feeling of safety among Ukrainian women remained even after controlling for socio-demographic and lifestyle factors (1.72; 1.13–2.62). This association was weaker than the corresponding association for Swedish women (1.97; 1.41–2.76). Among Swedish women and men, the associations between feeling of safety and depressive symptoms remained after controlling for other potential confounding factors, and this association was stronger for men compared to women for those with moderate level feeling of safety. Swedish women with low feeling of safety were twice as likely to report having depressive symptoms compared to women with high feeling of safety (1.97; 1.41–2.76) while Swedish men with low feeling of safety were four times more likely to report depressive symptoms (3.91; 2.19–6.97) compared to men with high feeling of safety. Swedish respondents who reported that they were “never alone after dark” also had higher odds for depressive symptoms among both women (1.95; 1.50–2.54) and men (3.21; 1.83–5.63).

The association between age and depressive symptoms differed in the two countries. In Sweden, the older you are the lower the odds for having depressive symptoms, while in Ukraine, the odds of having depressive symptoms were higher for the older age groups. Women and men older than 60 years in Ukraine had 8.66 (5.36–14.0) and 6.15 (2.89–13.1) higher odds for depressive symptoms respectively compared to men and women in the youngest age group 18–29. 

Adjusted odds ratio (with 95% confidence intervals) for depressive symptoms by levels of cognitive social capital for both sexes in Sweden and Ukraine are presented in Additional file 3: Table S3.

### Association between SRH, social capital, and socio-demographic and lifestyle factors

Additional file 2: Table S2 presents the crude ORs for poor SRH for women and men in both countries by level of social capital, socio-demographic, and lifestyle variables. Both institutional trust and feeling of safety were significantly associated with SRH in the Swedish context. Swedish women and men reporting low levels of institutional trust and feeling of safety were more likely to report poor SRH. In addition, there was a clear gradient such that low level of trust and feeling of safety increased the odds for poor SRH more compared to having moderate trust and feeling of safety. Swedish women and men with low levels of trust were more than twice as likely to report poor SRH compared to those with high levels of trust (2.52; 2.03–3.13 for women and 2.13; 1.76–2.59 for men). Similarly, low feeling of safety increased the odds of reporting poor SRH among women and men, and this association was stronger for men than women (2.41; 1.77–3.27 for women and 3.21; 1.84–5.61 for men). In addition, low level of institutional trust was associated with poor SRH for Ukrainian women (1.75; 1.12–2.73) while low feeling of safety was significantly associated with poor SRH for Ukrainian men (1.57; 1.04–2.36).

The adjusted odds ratios of having poor SRH by different levels of social capital for women and men in Sweden and Ukraine are presented in Table 5. Access to cognitive social capital was significantly associated with SRH in Sweden both for women and men even after adjusting for potential confounding socio-demographic and lifestyle factors. Consequently, having low access to institutional trust and feeling of safety increased the odds of having poor SRH for both women and men in Sweden. The association between low feeling of safety and poor SRH was weaker for women (2.15; 1.55–2.99) than for men (2.75; 1.58–4.80). In addition, there was a significant association between low level of institutional trust and poor SRH for Ukrainian women (1.88; 1.12–3.15), but no such association was found for Ukrainian men.

**Table 5:** Adjusted odds ratio (with 95 % confidence interval) for poor SRH by levels of cognitive social capital for women and men in Sweden and Ukraine

Variables	Sweden		Ukraine	
	Women	Men	Women	Men
Social capital				
Trust in the national government/parliament				
High	1***	1***	1**	1**
Moderate	1.50 (1.27–1.76)	1.26 (1.06–1.49)	1.29 (0.77–2.16)	1.24 (0.66–2.36)
Low	1.99 (1.58–2.50)	1.82 (1.48–2.24)	1.88 (1.12–3.15)	1.67 (0.90–3.12)
No opinion	1.77 (1.49–2.10)	1.26 (0.99–1.58)		
Feeling of safety				
High	1***	1***	1***	1***
Moderate	1.63 (1.35–1.98)	3.03 (2.14–4.28)	1.35 (0.81–2.24)	1.02 (0.64–1.63)
Low	2.15 (1.55–2.99)	2.75 (1.58–4.80)	1.44 (0.91–2.26)	1.20 (0.76–1.89)
Never alone	2.25 (1.80–2.81)	2.63 (1.64–4.23)		
Other variables				
Age				
18–29	1***	1***	1***	1***
30–59	1.50 (1.24–1.81)	1.88 (1.50–2.36)	5.55 (3.79–8.13)	4.46 (2.73–7.31)
60+	2.05 (1.65–2.55)	2.95 (2.29–3.80)	56.97 (29.1–111.7)	22.8 (11.4–45.6)
Education				
Short	1***	1***	1***	1***
Medium	0.78 (0.65–0.95)	0.69 (0.57–0.83)	0.73 (0.30–1.76)	0.62 (0.26–1.47)
Long	0.71 (0.60–0.84)	0.62 (0.51–0.74)	0.48 (0.20–1.17)	0.54 (0.22–1.28)
Marital status				
Living without a partner	1***	1***	1	1
Living with partner	0.93 (0.81–1.06)	0.72 (0.61–0.85)	1.28 (0.91–1.80)	1.37 (0.87–2.17)
Small children				
No	1***	1***	1***	1***
Yes	0.64 (0.52–0.80)	0.94 (0.75–1.19)	0.98 (0.61–1.57)	1.41 (0.70–2.86)
Smoking				
No	1***	1***	1***	1***
Yes	1.80 (1.53–2.10)	1.63 (1.35–1.97)	1.68 (0.91–3.09)	0.92 (0.62–1.37)
Alcohol ever				
No	1***	1***	1	1
Yes	0.51 (0.43–0.61)	0.62 (0.49–0.79)	2.18 (1.50–3.17)	1.85 (1.09–3.13)

The asterisks denote significant level of Chi Square test of p < 0.05 (*) or p < 0.01 (**) or p < 0.001 (***)

Age was associated with poor SRH in both countries and the older age groups were more likely of reporting poor SRH. Swedish women of the age 30–59 had 1.50 (1.24–1.81) higher odds for poor SRH compared to younger women and the corresponding figure for Ukrainian women was 5.55 (3.79–8.13). The same pattern was found for men I both countries; Swedish and Ukrainian men aged 30–59 had 1.88 (1.50–2.36) versus 4.46 (2.73–7.31) higher odds for poor SRH compared to younger men. Swedish and Ukrainian women above 60 years had 2.05 (1.65–2.55) versus 56.97 (29.1–111.7) higher odds for poor SRH compared to the youngest age groups and among men the corresponding figures were 2.95 (2.29–3.80) in Sweden versus 22.8 (11.4–45.6) in Ukraine.

Adjusted odds ratio (with 95% confidence intervals) for self-rated health by levels of cognitive social capital for both sexes in Sweden and Ukraine are presented in Additional file 3: Table S3.

## Discussion

### Summary of the main findings

The aim of this study was to analyze and compare associations between levels of individual cognitive social capital, SRH, and depressive symptoms among men and women in Sweden and Ukraine. The results show that low feeling of safety increases the odds for having poor SRH in Sweden both for women and men, even after adjusting for socio-demographic and lifestyle factors. In addition, a significant association between low level of institutional trust and poor SRH was found in Sweden for both sexes and for Ukrainian women. Further, our results show that lacking feeling of safety is significantly associated with depressive symptoms for men and women in Sweden, and for women in Ukraine, even after adjusting for potential confounding socio-demographic and lifestyle factors. No significant associations between cognitive social capital, SRH, and depressive symptoms were found for Ukrainian men after adjusting for other variables.

### Levels of cognitive social capital are higher in Sweden compared to Ukraine

The results show that institutional trust is considerably lower in Ukraine compared to Sweden which goes in line with findings from other studies. Other studies have also found a variation in levels of social capital between different nations. For example, the results of this research support the results of Sapsford and Abbot who found that among the countries that participated in their study about trust in post-communist societies, Ukraine stood out as the country with the least trust in the national government [54]. The collapse of the USSR in 1991 (of which Ukraine was a member) brought economic instability to the majority of the population and resulted in a lot of distrust in society. All post-communist countries have a history of totalitarian rule and forced participation in public affairs, and this has created distrust in public institutions and a retreat from the public sphere into the private [55]. In contrast, Sweden does not have such a history and has been characterized by a century of relative political stability [56] and this might explain the higher levels of institutional trust. Further, Pitchler and Wallace compared the patterns of social capital in Europe and concluded that Scandinavian countries had the highest levels of both formal social capital (trust) and informal social capital (social networks and social and family support), while in Eastern Europe informal social capital was more prominent over formal social capital [57]. A study comparing well-being and social capital across 142 countries also found that social support and social trust were higher in high-income countries compared to lower and middle-income countries [48]. However, one must bear in mind that in this study we measured trust in political institutions and not generalized social trust. It might well be, as suggested by others [55, 57], that a lack of institutional trust in former communist countries might encourage informal trusting social networks to develop.

### Cognitive social capital seems to be more important for depressive symptoms and self-rated health in Sweden than Ukraine

The results show that the association between feeling of safety and SRH and depressive symptoms is stronger in Sweden than in Ukraine. Cross-cultural studies have found consistent patterns of a positive association between individual social capital and SRH [54, 57] and have shown that these associations are often stronger in higher income countries [57]. However, these studies have used social support, trust, and volunteering as proxies for social capital, and we have not been able to find any cross-cultural studies on the association between feeling of safety and health. Our study indicates that there are cross-cultural differences in the association between feeling of safety and SRH.

One possible explanation for the results might be differences in public expectations in Sweden and Ukraine. Living in a post-socialist regime, such as Ukraine, people might not expect “the state” to protect them and thus they might need to cooperate informally in order to make efforts to “protect” themselves. Consequently, being able to feel safe might not be anything that is expected in general. On the contrary, in a welfare regime such as Sweden, the expectations for being able to feel safe might be higher.

With regards to trust, the results show an association between low levels of trust and poor SRH for both men and women in Sweden and for women in Ukraine. The study thus indicates that there might be gender and cultural differences in the associations between trust and SRH. The results are in line with those of Lindstrom who investigated the association between institutional trust and self-reported psychological health in the Skåne region in southern Sweden. He concluded that low trust in institutions such as the Swedish Parliament is significantly associated with poor psychological health [34]. Another study comparing the associations between social capital and SRH for men and women in six low- and middle-income countries also found variations in the association between trust and SRH [10]. While an association between trust and good SRH was found for women in most countries, this association was more complex and less robust for men across countries.

### Feeling of safety is more important for the health of men in Sweden

One important finding in this study is that feeling of safety seems to be more important for men’s self-rated and mental health compared to women in Sweden. This somewhat contradicts other studies that have found safety to be more important for the health of women than men. A qualitative study from Sweden explored what constitutes a health-enabling neighborhood for men and women, respectively, and found that women emphasized the importance of feeling of safety in the living environment more than men [58]. However, other cross-sectional studies from Australia [55, 56, 59] and Sweden [33] have found an association between neighborhood safety and good SRH for both men and women. One of the possible explanations for the gendered findings in our study might be the existing cultural constructions of femininity and masculinity in Western societies. According to traditional (hegemonic) constructions of masculinity, men in Sweden might be expected to be the protector (besides being the provider) and therefore might be expected to always feel safe. When not being able to live up to the expected masculinity norm—i.e. feeling safe—this might be harmful for self-rated and mental health. Due to socially constructed femininities, women are not expected to be able to feel safe, and this is why feeling of not being safe might be less harmful for their (self-perceived) health compared to men. The lack of association between feeling of safety and health among Ukrainian men might indicate that even if they report feeling unsafe it is not harmful for their health because the general expectations for feeling safety might be lower in Ukrainian society. In addition, cultural constructions of femininity and masculinity might differ between cultural contexts such as Sweden and Ukraine.

### Strengths and limitations

A major strength of this study is the cross cultural comparison because nationwide comparisons about the associations between social capital and health are still to a large extent lacking. Another strength is the sex-stratified analysis that gives more information about the complex associations between cognitive social capital and SRH and depressive symptoms.

This study also has some limitations. One limitation is the cross-sectional design, which does not allow us to draw any causal inference conclusion for the association between depressive symptoms and self-rated health with social capital. The associations we observed in this study might reflect that people with lower social capital were at higher odds of reporting depressive symptoms. It might also reflect the reverse causality, as people with depressive symptoms might have different access to social capital, and even if they had the same access, they might have different perceptions on their social capital level. Use of longitudinal panel data would allow us to investigate the causal association further.

Another limitation concerns the use of two different datasets that included slightly different questions, which limits the ability to make exact comparisons. The nationally representative datasets, however, do enable national comparisons. A further possible limitation is the use of secondary register data that does not fully allow for the proper tracking of missing data and as a result reduces the sample size.

## Conclusions

In general, cognitive social capital in the form of institutional trust and feeling of safety is higher in Sweden than in Ukraine, and there is a stronger association between cognitive social capital and SRH in Sweden than in Ukraine. Institutional trust seems to be more important for SRH in men and women in Sweden compared to Ukraine. Living in a high-trust society (Sweden) but having low trust oneself seems to be harmful for SRH for men and women in Sweden, while living in a low- trust society (Ukraine) and having low trust does not seem to be harmful for SRH of Ukrainian men.

A lack of feeling of safety is harmful for both SRH and depressive symptoms in Sweden, and the effect is greater among men.

This study shows the complexity of the links between institutional trust and feeling of safety and SRH and depressive symptoms. It also provides evidence that cognitive social capital and its association with self-rated and mental health for men and women differs between a post-Soviet transitional country and a Western welfare state. Interventions aiming to increase trust and feeling of safety for health-promoting purposes might be effective in the Swedish context while this is less likely to be the case in the Ukrainian context.

## Additional files

10.1186/s13033-016-0068-4 Crude odds ratios with 95 % confidence intervals for depressive symptoms for women and men in Sweden and Ukraine by levels of social capital and socio-demographic and lifestyle factors.
10.1186/s13033-016-0068-4 Crude odds ratios with 95 % confidence intervals for poor SRH for women and men for Sweden and Ukraine.
10.1186/s13033-016-0068-4 Adjusted odds ratio (with 95 % confidence intervals) for depressive symptoms and self-rated health by levels of cognitive social capital for both sexes in Sweden and Ukraine.

## Abbreviations

WHO: World Health Organization
SRH: self-rated health
WHS: World Health Survey
SNPHS: Swedish National Public Health Survey
