# Supplementary material for: Gender differences in the association between cognitive social capital, self-rated health, and depressive symptoms: a comparative analysis of Sweden and Ukraine
Source: Int J Ment Health Syst. 2016 May 4;10:37. doi: 10.1186/s13033-016-0068-4 (PMC4855473; doi:10.1186/s13033-016-0068-4)
Supplement: Supplementary file 1 — 10.1186/s13033-016-0068-4 Crude odds ratios with 95 % confidence intervals for depressive symptoms for women and men in Sweden and Ukraine by levels of social capital and socio-demographic and lifestyle factors. [file 13033_2016_68_MOESM1_ESM.docx]

Supplement 1

Table S1. Crude odds ratios with 95% confidence intervals for depressive symptoms for women and men in Sweden and Ukraine by levels of social capital and socio-demographic and lifestyle factors*.*

| ***Variables*** | | | **Sweden** | | | | | **Ukraine** | | | |
| --- | --- | --- | --- | --- | --- | --- | --- | --- | --- | --- | --- |
|  | | | **Women** | | | **Men** | | **Women** | | **Men** | |
| ***Social capital*** | | | | | | | | | | | |
| Trust in the national government/  parliament | High | 1 | | 1 | | | 1 | | 1 | | |
|  | Moderate | 1.02 (0.86–1.20) | | 1.03 (0.84–1.27) | | | 0.97 (0.63–1.51) | | 1.40 (0.65–2.98) | | |
|  | Low | 1.25 (0.98–1.59) | | 1.44 (1.14–1.83) | | | 1.41 (0.93–2.12) | | 1.55 (0.76–3.14) | | |
|  | No opinion | 1.03 (0.86–1.24) | | 1.04 (0.80–1.37) | | |  | |  | | |
| Feeling of safety | High | 1 | | 1 | | | 1 | | 1 | | |
|  | Moderate | 1.65 (1.35–2.01) | | 2.93 (2.08–4.16) | | | 1.04 (0.68–1.58) | | 1.02 (0.59–1.77) | | |
|  | Low | 2.19 (1.59–3.01) | | 4.10 (2.32–7.24) | | | 1.62 (1.12–2.35) | | 1.46(0.90–2.36) | | |
|  | Never alone | 1.22 (0.96–1.54) | | 2.07 (1.24–3.48) | | |  | |  | | |
| ***Other variables*** | | | | | | | | | | | |
| Age | 18–29 | 1 | | 1 | | | 1 | | 1 | | |
|  | 30–59 | 0.56 (0.48–0.66) | | 0.78 (0.64–0.96) | | | 2.53 (1.70–3.76) | | 1.74 (0.86–3.51) | | |
|  | 60+ | 0.27 (0.22–0.34) | | 0.40 (0.31–0.52) | | | 8.90 (5.79–13.7) | | 6.91 (3.38–14.1) | | |
| Education | Short | 1 | | 1 | | | 1 | | 1 | | |
|  | Medium | 1.63 (1.36–1.98) | | 1.52 (1.19–1.92) | | | 0.30 (0.19–0.45) | | 0.25 (0.14–0.45) | | |
|  | Long | 1.51 (1.25–1.82) | | 1.35 (1.06–1.71) | | | 0.20 (0.13–0.31) | | 0.31 (0.17–0.58) | | |
| Marital status | Living without a partner | 1 | | 1 | | | 1 | | 1 | | |
|  | Living with partner | 0.68 (0.59–0.78) | | 0.60 (0.50–0.71) | | | 0.70 (0.54–0.90) | | 1.48 (0.94–2.32) | | |
| Small children | No | 1 | | 1 | | | 1 | | 1 | | |
|  | Yes | 1.30 (0.09–1.58) | | 1.42 (1.14–1.78) | | | 0.78 (0.52–1.16) | | 0.80 (0.32–1.98) | | |
| Smoking | No | 1 | | 1 | | | 1 | | 1 | | |
|  | Yes | 1.17 (1.00–1.39) | | 1.63 (1.31–2.03) | | | 0.68 (0.41–1.12) | | 0.66 (0.43–1.00) | | |
| Alcohol ever | No | 1 | | | 1 | | 1 | | | | 1 |
|  | Yes | 1.03 (0.85–1.26) | | 0.85 (0.63–1.13) | | | 1.04 (0.79–1.37) | | 0.67 (0.41–1.11) | | |
