# Supplementary material for: Gender differences in the association between cognitive social capital, self-rated health, and depressive symptoms: a comparative analysis of Sweden and Ukraine
Source: Int J Ment Health Syst. 2016 May 4;10:37. doi: 10.1186/s13033-016-0068-4 (PMC4855473; doi:10.1186/s13033-016-0068-4)
Supplement: Supplementary file 2 — 10.1186/s13033-016-0068-4 Crude odds ratios with 95 % confidence intervals for poor SRH for women and men for Sweden and Ukraine. [file 13033_2016_68_MOESM2_ESM.docx]

Supplement 2

Table S2. Crude odds ratios with 95% confidence intervals for poor SRH for women and men for Sweden and Ukraine

| ***Variables*** | | | | **Sweden** | | | | **Ukraine** | | | |
| --- | --- | --- | --- | --- | --- | --- | --- | --- | --- | --- | --- |
|  | | | | **Women** | | **Men** | | **Women** | | | **Men** |
| ***Social capital*** | | | | | | | | | | | |
| Trust in the national government/  parliament | High | | 1 | | 1 | | 1 | | | 1 | |
|  | Moderate | | 1.64 (1.40–1.92) | | 1.36 (1.16–1.61) | | 1.14 (0.72–1.83) | | | 0.94 (0.53–1.26) | |
|  | Low | | 2.52 (2.03–3.13) | | 2.13 (1.76–2.59) | | 1.75 (1.12–2.73) | | | 1.37 (0.80–2.35) | |
|  | No opinion | | 2.27 (1.93–2.68) | | 1.56 (1.26–1.94) | |  | | |  | |
| Feeling of safety | High | | 1 | | 1 | | 1 | | | 1 | |
|  | Moderate | | 1.67 (1.39–2.01) | | 3.53 (2.55–4.87) | | 1.28 (0.80–2.04) | | | 1.09 (0.72–1.65) | |
|  | Low | | 2.41 (1.77–3.27) | | 3.21 (1.84–5.61) | | 1.44 (0.96–2.15) | | | 1.57 (1.04–2.36) | |
|  | Never alone | | 3.79 (3.10–4.65) | | 4.35 (2.73–6.93) | |  | | |  | |
| ***Other variables*** | | | | | | | | | | | |
| Age | | 18–29 | 1 | | 1 | | 1 | | 1 | | |
|  |  | 30–59 | 1.45 (1.22–1.72) | | 1.64 (1.34–2.02) | | 5.59 (3.92–7.95) | | 5.00 (3.24–7.71) | | |
|  |  | 60+ | 2.93 (2.44–3.53) | | 3.03 (2.44–3.77) | | 49.1 (25.3–95.1) | | 26.0 (13.8–49.0) | | |
| Education | | Short | 1 | | 1 | | 1 | | 1 | | |
|  |  | Medium | 0.44 (0.38–0.52) | | 0.46 (0.39–0.55) | | 0.15 (0.07–0.33) | | 0.28 (0.14–0.56) | | |
|  |  | Long | 0.42 (0.36–0.48) | | 0.45 (0.38–0.54) | | 0.10 (0.04–0.22) | | 0.22 (0.11–0.45) | | |
| Marital status | | Living without a partner | 1 | | 1 | | 1 | | 1 | | |
|  |  | Living with partner | 0.82 (0.73–0.93) | | 0.87 (0.76–1.00) | | 1.12 (0.84–1.49) | | 2.90 (2.0–4.21) | | |
| Small children | | No | 1 | | 1 | | 1 | | 1 | | |
|  |  | Yes | 0.49 (0.41–0.60) | | 0.71 (0.58–0.88) | | 0.52 (0.35–0.76) | | 0.82 (0.49–1.50) | | |
| Smoking | | No | 1 | | 1 | | 1 | | 1 | | |
|  |  | Yes | 1.86 (1.60–2.15) | | 1.85 (1.54–2.22) | | 1.11 (0.66–1.89) | | 0.88 (0.63–1.23) | | |
| Alcohol ever | | No | 1 | | 1 | | 1 | | 1 | | |
|  |  | Yes | 0.43 (0.36–0.50) | | 0.50 (0.40–0.62) | | 1.47 (1.08–1.99) | | 1.50 (0.96–2.33) | | |
