# Supplementary material for: Gender differences in the association between cognitive social capital, self-rated health, and depressive symptoms: a comparative analysis of Sweden and Ukraine
Source: Int J Ment Health Syst. 2016 May 4;10:37. doi: 10.1186/s13033-016-0068-4 (PMC4855473; doi:10.1186/s13033-016-0068-4)
Supplement: Supplementary file 3 — 10.1186/s13033-016-0068-4 Adjusted odds ratio (with 95 % confidence intervals) for depressive symptoms and self-rated health by levels of cognitive social capital for both sexes in Sweden and Ukraine. [file 13033_2016_68_MOESM3_ESM.docx]

Table S3. Adjusted odds ratio (with 95% confidence intervals) for depressive symptoms and self-rated health by levels of cognitive social capital for both sexes in Sweden and Ukraine.

| ***Variables*** | | | **Sweden** | | | | **Ukraine** | | | |
| --- | --- | --- | --- | --- | --- | --- | --- | --- | --- | --- |
|  | | | **LRD** | | **SRH** | | **LRD** | | **SRH** | |
| ***Social capital*** | | | | | | | | | | |
| Trust in the national government/  parliament | High | 1* | | 1*** | | 1*** | | 1** | | |
|  | Moderate | 1.09 (0.96–1.24) | | 1.40 (1.25–1.56) | | 1.13 (0.76–1.69) | | 1.25 (0.83–1.88) | | |
|  | Low | 1.25 (1.06–1.49) | | 1.90 (1.64–2.20) | | 1.33 (0.92–1.94) | | 1.77 (1.18–2.64) | | |
|  | No opinion | 1.01 (0.87–1.18) | | 1.56 (1.37–1.78) | |  | |  | | |
| Feeling of safety | High | 1*** | | 1*** | | 1*** | | 1*** | | |
|  | Moderate | 1.83 (1.54–2.17) | | 1.91 (1.63–2.25) | | 0.98 (0.68–1.42) | | 1.15 (0.80–1.63) | | |
|  | Low | 2.37 (1.79–3.13) | | 2.19 (1.67–2.87) | | 1.50 (1.08–2.07) | | 1.29 (0.93–1.80) | | |
|  | Never alone | 2.06 (1.64–2.59) | | 2.37 (1.95–2.88) | |  | |  | | |
| ***Other variables*** | | | | | | | | | | |
| Sex | Women | 1*** | | 1*** | | 1*** | | 1*** | | |
|  | Men | 0.71 (0.63-0.80) | | 0.93 (0.84-1.03) | | 0.48 (0.36-0.64) | | 0.48 (0.36-0.64) | | |
| Age | 18–29 | 1*** | | 1*** | | 1*** | | 1*** | | |
|  | 30–59 | 0.76 (0.66–0.86) | | 1.68 (1.46–1.93) | | 2.41 (1.65–3.53) | | 4.93 (3.64–6.67) | | |
|  | 60+ | 0.37 (0.31–0.45) | | 2.42 (2.06–2.85) | | 7.31 (4.87–11.0) | | 35.3 (21.9–56.8) | | |
| Education | Short | 1*** | | 1*** | | 1*** | | 1*** | | |
|  | Medium | 1.14 (0.97–1.35) | | 0.73 (0.64–0.83) | | 0.59 (0.40–0.87) | | 0.65 (0.34–1.21) | | |
|  | Long | 1.28 (1.09–1.50) | | 0.67 (0.60–0.76) | | 0.52 (0.34–0.79) | | 0.49 (0.26–0.93) | | |
| Marital status | Living without a partner | 1*** | | 1*** | | 1*** | | 1*** | | |
|  | Living with partner | 0.71 (0.63–0.79) | | 0.86 (0.78–0.96) | | 0.85 (0.66–1.09) | | 1.25 (0.95–1.65) | | |
| Small children | No | 1*** | | 1*** | | 1** | | 1*** | | |
|  | Yes | 1.33 (1.15–1.55) | | 0.74 (0.63–0.86) | | 1.41 (0.92–2.16) | | 1.12 (0.75–1.69) | | |
| Smoking | No | 1*** | | 1*** | | 1*** | | 1*** | | |
|  | Yes | 1.26 (1.10–1.44) | | 1.70 (1.51–1.91) | | 0.88 (0.60–1.27) | | 1.05 (0.75–1.47) | | |
| Alcohol ever | No | 1 | | 1*** | | 1** | | | | 1 |
|  | Yes | 0.96 (0.81–1.13) | | 0.57 (0.50–0.65) | | 1.26 (0.94–1.68) | | 2.07 (1.52–2.81) | | |

The asterisks denote significant level of Chi-Square test of p<0.05 (*) or p<0.01 (**) or (***) p<0.001
